# The Condition of the Dentine in Pulpless Teeth

**Published:** 1893-03

**Authors:** W. C. Barrett

**Affiliations:** Buffalo, N.Y.


### The Condition of the Dentine in Pulpless Teeth.

BY W. C. BARRETT, M.D., D.D.S., OF BUFFALO, N.Y.

Read in the Section of Oral and Dental Surgery, at the Forty-third Annual Meeting of the American Medical Association, held at Detroit, Mich., June, 1892.

I had promised the Secretary of this Section that I would prepare and read a paper at this meeting upon the "Condition of the Dentine in Pulpless Teeth." It was a subject upon which I had bestowed some thought, but with the literature of which I had not made myself thoroughly acquainted. I had read Prof. Miller's work on "The Micro-Organisms of the Human Mouth," and his communications to the various journals, notably *The Dental Cosmos*, but not with this special subject in mind. I had not collocated his writings on this theme, and hence had not collated them. When this had been done I was astonished at the thorough manner in which the ground had been covered. Had I examined my subject earlier, I should not have attempted an excursion in the field, but having engaged to present this paper, I will endeavor to redeem my promise, premising that I can do little save to call attention to the great work done by Prof. Miller in this direction, a work which does not seem to be fully appreciated, and which, perhaps, will not be until each attempts, like me, especially to study it for himself.

In view of his exhaustive experiments, one can but wonder at the seemingly crude theories now held and taught by some men to whom we naturally turn for illumination. Perhaps I shall do more good by placing Dr. Miller's comprehensive studies before you for discussion than I would by effort in any other direction, for it must be remembered that his are not mere generalizations, such as mine must necessarily be; they are the result of long series of conclusive, scientifically conducted, original experiments and observations, and they are not to be overthrown by any inductive reasoning whatever. In view of the overwhelming original testimony which he introduces, no man has the right to dispute his deductions until he is fortified by a like mass of opposing facts, deduced from like carefully conducted experiments; or until he can indisputably demonstrate that the conclusions reached by Dr. Miller are not warranted by the results obtained.

The dentine, deriving its nutriment from the pulp of the tooth through the dentinal fibrillæ, is separated from all the other tissues of the body, through investment by the cementum and the enamel. The latter tissue is not only practically devoid of living matter, but it is not in direct relation with any tissues save the dentine itself. For our present purposes then, the enamel may be looked upon as a simple protecting cap, whose office it is to preserve the dentine and pulp from injury.

The cementum is of a different character. It is but slightly differentiated from true bone. It is in relation with a periosteal membrane, and it is nourished like bone through a system of lacunal corpuscles, connected by canaliculi.

In young teeth it is not infrequently the case that there is even a kind of system of Haversian canals, traversing the cementum and dentine, between the pericementum and pulp, whose office it is to assist in maintaining the vascular supply, and incidentally, perhaps, to afford nutriment to the deeper cementum corpuscles.

The dentine is yet more differentiated, and in its structure has lost the lacunal corpuscles, while the canaliculi are modified into the dental fibrillæ. Their office is not materially changed, nor are we to suppose that the contents of the dental tubuli differ very widely from the canaliculi of true bone. They form the living portion of the dentine, as the lacunal corpuscles and the canaliculi form that of true bone. The dentinal fibrillæ are in connection with the vascular pulp, and from it derive the nutriment necessary to their continued life. The corpuscles found in true bone and in cementum are absent, and hence there is little that bears a resemblance to that concentric texture of true bone which forms its lamellated structure. At the extremities of the dentinal fibrillæ, we have the irregular inter-tissual spaces which mark the boundary line between dentine and cementum, and which may, perhaps, be considered as modifications of the cementum corpuscles, but they are not intimately connected with the dentinal structures.

The dentinal fibrillæ, depending as they do on the vascular pulp for nutrition, certainly lose their protoplasmic vitality when separated from it. Let us consider for a moment the condition of the dentine in a tooth the pulp of which has, without medication, just been removed. The fibrillæ have been freshly torn from their relations with the pulp, and remain filling the dental tubuli as before their violent separation. Like all protoplasmic material, they are composed of the great quaternary of elements which enter into the composition of all organic elementary structures.

But the dentinal fibrillæ probably also contain other elements, such as sulphur and phosphorus, which make it more nearly the nature of albumen. Indeed, it is probable that if we consider it as clearly albuminous, we shall not do much violence to nature. Carpenter says that this substance is the proper pabulum of the animal tissues generally, and so the dental fibrillæ, possibly modified somewhat, may be looked upon as albuminous in their nature.

The dentinal tubules which contain the fibrillæ are exceedingly minute. For the putrefaction of the contents of the tubuli it would be essential that putrefactive organisms should enter them. While it is quite possible that these may penetrate the tubuli, it must be to but a limited extent. In an article in the *Dental Cosmos*, for May, 1890, upon "The Decomposition of the Contents of the Dental Tubuli," Dr. Miller shows that from their minute size and the very small amount of nutriment for the micro-organisms which they contain, any bacteria which enter them must soon perish from want of pabulum, to say nothing of the fact that they are mostly aerobic, and so could not possibly live in the tubuli for any length of time.

But not satisfied with *a priori* reasoning, he commences a rather remarkable series of experiments and observations, from which he incontestably deduces the conclusion that bacteria do not penetrate the tubuli of undecayed dentine to any marked extent; certainly not enough to cause any necessity for the disinfection of the dentinal substance in teeth in which there has been no decay within the pulp canals.

I need not enlarge upon this. The record is open to the inspection of all, and no one has yet dared to take issue with Dr. Miller upon this point. It must be accepted that in the dentine of the roots of the teeth in which decay has made no progress, there is no infection of dentine that demands any antiseptic treatment aside from that usual in pulp canals.

What then becomes of the contents of the dentinal tubuli? It is but necessary to consider their character to form a clear estimate of this. The dentinal fibers cannot be considered as pure albumen, for that is only found in connection with other substances in the body. But whatever its exact composition, it presents certain characteristics, one of which is its tendency, when at rest and separated from living tissue, to dissociate the watery portion, and to form a dried residuum which is insoluble. This condition may also be produced by most acids, by the metallic salts and by creosote.

The natural tendency then, for an albuminoid fibril, would be to assume a mummified condition, most conducive to its indefinite preservation, when kept in the state in which it will be found in the dentine of a tooth root.

Fibrine is but comparatively slightly differentiated from albumen. It contains the same elements, but it offers certain physical characteristics that distinguish it. Albumen is coagulated by heat, and by certain agents. Fibrine coagulates spontaneously when exposed to the air. If then the fibrillæ of the dentine approach fibrine in their characteristics they will undergo the change called coagulation without the intervention of any medicinal agent whatever. It is in this condition that, if sealed up from external influences, they present the very best state for the preservation of the tissue in which they are found. Hence the condition of the dentine is best in which the contents of the tubuli have been coagulated, or separated from the watery elements, and permitted to become a kind of minute mummy within their investing sheaths.

But the investigations of Miller, to which I have already referred, determine another matter. He did not find that the mouths of the dental tubuli, in teeth from which the pulp had been removed, were opened toward the pulp chamber.

He had under examination nineteen teeth. In ten of the roots of them he found the canals to be completely or partially lined with a homogeneous structure, containing few or no tubuli, but occasionally a formation resembling a bone lacuna, and bearing a much nearer resemblance to cementum than to dentine. This layer is impenetrable to micro-organisms, and therefore forms a protection to that tissue when the pulp has been removed. We find, then, that the dentine is not only protected by the cementum on its external aspect, but very often by a modification of this tissue on its inner surface.

There is, therefore, little danger of infection of the contents of the dental tubuli. But even if this were possible, I cannot conceive that it would matter much. The amount of infectious matter would be so small, and the chance for the penetration of the gaseous products of decomposition through the dentinal tubuli, past the zone of demarcation between dentine and cementum, and finally through the cementum corpuscles and canaliculi, which are themselves filled with living matter, would be so remote, and its influence upon the pericementum so infinitesimally small, that it seems folly to take it into consideration in the presence of so much more potent factors.

Clinical experience, too, as is so plainly stated by Dr. Miller in the article to which reference has been made, does not sustain the theories of those who insist upon the possibility of infection from dentine. If the contents of the pulp canal be completely removed, the territory made thoroughly aseptic and the space finally filled, there is, so far as I am aware, no record of any trouble from the dentine. Even admitting that there exists within the dental tubuli, matter which is susceptible to putrefactive changes, it is too small in amount to induce any disturbing conditions in the presence of a pulp canal carefully filled with a material which must seal the exceedingly minute mouths of the tubules wherever they are not already stopped by the modified dentinal layer, of which Miller speaks. In the presence of much more important things, it seems like bad judgment to elevate such a mole-hill into a mountain, for it has a tendency to carelessness in things that need more attention. I doubt whether there has ever been a case of re-infection of the tissues about a tooth that has had its root filled after antiseptic treatment, that cannot be directly traced to some imperfection in the manipulation, or to some infected pockets outside the tooth itself. It is well known that the usual place for the formation of an infected pocket is at or near the apex, and that there may be a penetration of the micro-organisms, and the formation of infected pockets for some distance back in the bone. I have myself seen at least three such, extending back from the point of an incisor tooth until the last one was over the second bicuspid, and each of these separate pockets connected with the original one at the apex of the incisor.

They were only reached by a long incision through the gum and external alveolus, extending from one to the other until the last one had been reached. If the infection had been from the dentine, it would have manifested itself quite differently.

Undoubtedly there are cases in which the infected spot is at some point in the pericementum between the gingival margin and the apex of the root. But this does not by any means prove that it came from infected dentine. Perhaps, after the apical foramen, the most usual place for the formation of an abscess is at the bifurcation of the roots of a bicuspid or molar. I believe this to be due to the fact mentioned earlier in this paper, that something analogous to an Haversian canal there may penetrate the cementum and dentine, from the pericementum to the pulp. I have demonstrated such many times.

Not infrequently an abscess refuses to yield to the usual treatment through the pulp canal, and for the reason that the medicaments fail to reach the infected point. But such instances cannot be said to be produced by infected dentine.

There is, in truth, another foramen analagous to that at the apex, and it becomes septic in precisely the same manner as does the latter.

In what I have previously urged, we have presupposed that caries has not commenced within the pulp canal. And yet we are all well aware that in cases in which the pulp had been long dead, and there has been extensive decay of the coronal portion of the tooth, there was caries of the dentine commencing within the pulp chamber and extending down the canal.

In instances like this, of course, there will be thorough infection of the dentine. It will not, however, be due to decomposition of the contents of the dental tubuli, but to infectious matter which may find its way into the hollow from external sources. It would be as manifestly improper to fill such a canal without removing the carious portion, as it would be to fill a cavity from the outside without careful excavation.

Here would be an instance in which the reaming out of the canal to the depth to which caries had penetrated would be eminently proper practice. It is very seldom that this decay will extend far down the root canal, or be very deep, without such destruction of the crown and cervical portions of the root as to make preservation practically impossible.

As regards the use of coagulating agents in the treatment of root canals, I cannot conceive of any objection to them. If by any it be thought necessary that dentine should be disinfected and made more aseptic, this can only be accomplished by agents which have the power of penetration in a marked degree. These are usually coagulants, like chloride of zinc, bichloride of mercury, etc. Upon this subject I can only refer to the experiments and observations reported by Dr. Miller, in an article on "The Relative Rapidity with which various Antiseptics Penetrate Pulp Tissue," published in the *Dental Cosmos* for May, 1891.

### DISCUSSION.

Dr. E. L. Clifford said the discoloration found in dead teeth was probably caused by the formation of sulphides of some of the different metallic agents used in medicaments. The less we treat the teeth in the vast majority of cases, the better will be the results. No law can be laid down to which exceptions could not be found, but in the majority of cases, the quicker we fill a root canal and the cavity over it, the more satisfactory the operation will be. Two things, however, work against immediate root-filling: first, the difficulty of drying the root; and second, soreness of the tissues about the root. If there is no way to dry it thoroughly, or if it is too sore to work on, then it is better to wait. In all favorable cases he advocated immediate filling.

Dr. W. W. Allport said the section was under obligation to Dr. Barrett for a most excellent paper.

In our practice we have two classes of pulpless teeth to treat. Those we devitalize, leaving the dentinal fibers in the condition when devitalization took place, are in the most favorable condition for successful treatment. The other class is where the nerve has been dead for a long time. Here the condition is different, and the treatment must be adapted to the altered condition.

Our aim should be to prevent discoloration of the dentine. Whether this can best be done by the use of oils or of chlorine, or by thoroughly drying the cavity and canal, so as to leave the fibers in a mummified condition that will prevent decomposition, is the question to decide. In old cases we should use every means in our power to restore the normal color, but in freshly devitalized teeth little or no treatment is necessary.

Dr. Gish said that whatever treatment was necessary could as well be done in one sitting as in twenty, and that the duty of the dentist to his patient calls for the course of treatment necessitating the least trouble and pain, therefore he advocated immediate filling.

Dr. M. H. Fletcher said that to him it seemed that the pulp-canal, before filling, must be surgically clean; that is, it should have all substances removed which might contain or support any spores or bacteria. In a case reported by Dr. H. A. Smith in a paper read before the Mississippi Valley Dental Society, trouble arose at the apex of a tooth that had been filled for fourteen or fifteen years. In the discussion it was said that there had been a continual effort at the apex of the root to dissolve the root as a foreign material. This will always happen if there should be an excess of filling-material forced through the end of the root, and if the tubuli had not been thoroughly disinfected the septic matter contained in them would set up suppuration.

In regard to the substance covering the tubules from the pulp chamber, he had learned that after the tooth is fully formed there is a deposit, as it has been called, a translucent zone, lining the chamber and pulp-canal. But the deposit, as he had seen it, is penetrated by the tubuli. This must be so or the deposit could not take place, and he thought the tubuli were sufficiently numerous to need disinfection.

Dr. G. L. Curtis said the paper was an argument in favor of removing the pulp while it was healthy, and not destroying it by any substances that would enter the tubules. He could understand how in teeth so treated the deposit spoken of would occur, but in teeth dead for a long time it would not be there.

Dr. A. E. Baldwin did not agree with Dr. Barrett's idea, that anything was so well proven that a dentist should finally accept it as a truth without satisfying himself by independent investigation. Because another had formed a theory, and by experiment satisfied himself of the truth of it, was no reason anyone else should accept it. In regard to bacteria and microbes, and the necessity of making our root-canals aseptic, he disagreed with the paper. He did not think he could get them in better condition than by desiccation. He seldom uses anything in the way of antiseptics except carbolic acid in five-per cent. solution, and has very good success. If he can get the root cleaned out in an ordinary sense, whether it is "surgically clean" or not, and get it thoroughly dry, he did n t worry about the success of the filling. He thought a great deal of the irritation about the end of the root could be treated from the outside rather than through the root-canal.

Dr. Barrett said we would have to modify our view in regard to the pathology of alveolar abscess. He believed it to be thoroughly demonstrated that the pericementum furnishes all blood-supply to the growing tooth. He did not believe anybody ever saw arteries and veins passing through the apical foramen. He believed there is something like Haversian canals that pass through the dentine and the cementum to the pericementum, and that pus will often be found about diseased teeth at the bifurcation of the roots, and this shows why the treatment through the roots will not succeed. He believes that thorough drying will make a tooth aseptic, but sometimes the point of infection will be found away off from the tooth that seems to be the cause of suffering to the patient. In these cases, of course, the treatment must be on the outside, and followed up till the point of infection is discovered and broken up and cleaned out.

